# Relationship Between Smoking and Lipid Profile in Four Primary Health Care Units: A Research Study

**DOI:** 10.7759/cureus.69172

**Published:** 2024-09-11

**Authors:** Inês R Sousa, Marlene Miranda, Hugo Gomes, Adriana Figueiredo, Jessica Silva, Juliana Campos

**Affiliations:** 1 Family Medicine, Unidade de Saúde Familiar Viatodos, Barcelos, PRT; 2 Family Medicine, Unidade de Saúde Familiar Lígios, Barcelos, PRT; 3 Family Medicine, Unidade de Saúde Familiar Calécia, Barcelos, PRT; 4 Family Medicine, Unidade de Saúde Familiar Santo António, Barcelos, PRT

**Keywords:** atherosclerosis, cardiovascular disease, dyslipidemia, risk factors, smoking

## Abstract

Introduction

Smoking is the most preventable cause of cardiovascular disease (CVD). Dyslipidemia is also an important risk factor for CVD. Yet, research has provided contradicting findings regarding the association between smoking and blood lipids. This study aims to assess the relationship between smoking and dyslipidemia, as well as compliance with the previously established treatment target.

Materials and methods

This was a cross-sectional study. Apparently, healthy users aged 40 or over, were from four primary health care units (PHCU) in Portugal. The inclusion criteria were: age between 40 and 69 and active enrollment in one of the four PHCU. The exclusion criteria were: background of atherosclerotic disease, heart failure, chronic kidney disease, diabetes mellitus, and a history of acute myocardial infarction; no record of lipid profile or triglyceride value of more than 400 mg/dL. Cardiovascular risk levels were determined using the Systematic Coronary Risk Evaluation (SCORE) 2 risk. The low-density lipoprotein cholesterol (LDL-c) target was considered the recommendation by the European Society of Cardiology/European Atherosclerosis Society (ESC/EAS) 2019.

Results

From an initial population of 16,939 patients, 12,076 apparently healthy patients were included. The difference between cholesterol values is statistically significant, with total cholesterol, LDL-c, and triglyceride values tending to be higher in the smoking group and the high-density lipoprotein cholesterol (HDL-c) value tending to be higher in the non-smoking group. Regarding the LDL target, there is also a statistically significant difference between smokers and non-smokers.

Discussion

Smoking and dyslipidemia are two isolated cardiovascular risk factors. Their association has been questioned. This study demonstrated that there is an association between smoking and the lipid profile, but also with the LDL target. However, there are some biases that must be considered, and which do not allow for generalization.

Conclusions

This study concludes that smoking negatively influences the lipid profile, with lipids being found less frequently on target. However, more studies are needed in this area, particularly studying other relevant factors such as body mass index.

## Introduction

Cardiovascular diseases (CVDs) are the leading cause of death in Portugal, accounting for 32.5% of disease-related deaths in 2021. These results align with international statistics, which estimate that CVDs are responsible for one-third of all deaths worldwide [1]. This group of pathologies results from the development of an atherosclerotic process over the years, associated with multiple cardiovascular risk factors. The most common risk factors include dyslipidemia, hypertension, diabetes mellitus, obesity, and smoking, conditions with a high prevalence both globally and nationally.

These risk factors are often interlinked, given that the pathophysiology of each individual condition can contribute to the development of the other conditions. Among these relationships, and taking into account the purpose of this work, the relationship between smoking and the development of dyslipidemia stands out.

Tobacco use increases the risk of all-cause mortality, being responsible for 50% of all avoidable deaths in smokers, with half of these due to atherosclerotic cardiovascular disease [2-3]. Worldwide, after high systolic blood pressure, smoking is the leading risk factor for disability-adjusted life-years [4]. According to data from the World Health Organization, in 2020, 22.3% of the global population were smokers, of which 36.7% were men and 7.8% were women [5]. On the other hand, the results of the 2019 National Health Survey showed that 17.0% of the population living in Portugal, aged 15 or over, were smokers (23.9% men and 10.9% women), with a ratio of 2.0 men for every woman [6].

This study aims to assess the relationship between smoking and dyslipidemia, as well as compliance with the previously established target.

## Materials and methods

This study was carried out in four primary health care units (PHCU) in Portugal (Unidades de Saúde Familiar Calécia, Lígios, Santo António and Viatodos). This is a cross-sectional study involving a population of apparently healthy users aged 40 or over, enrolled in the units under study.

Patients with the following inclusion criteria were considered: (i) age between 40 and 69; and (ii) active enrollment in one of the four PHCUs under study. The following were excluded: (i) those with atherosclerotic disease, heart failure, chronic kidney disease, diabetes mellitus, and a history of acute myocardial infarction; (ii) users with no record of total cholesterol, high-density lipoprotein cholesterol (HDL-c) and/or triglycerides or with a triglyceride value of more than 400 mg/dL.

The following data was collected from the selected sample: gender, age, tobacco consumption, last record of total cholesterol, HDL-c, triglycerides, and active problems listed in the exclusion criteria. Cardiovascular risk levels were determined using the Systematic Coronary Risk Evaluation (SCORE) 2 risk estimation methods [7] and low-density lipoprotein cholesterol (LDL-c) was calculated using the Friedewald formula [8]. Smoking status, classified as smoker or non-smoker, was assessed by a written record in an electronic medical file, obtained through an interview. To assess the achievement of the LDL-c target, the targets recommended by the European Society of Cardiology/European Atherosclerosis Society (ESC/EAS) 2019 [7] were considered: ≤116 mg/dL for low-risk; <100 mg/dL for moderate risk; <70 mg/dL for high risk; and <55 mg/dL for very high risk.

The data was analyzed in an anonymized database using IBM SPSS Statistics software (version 29.0, IBM Corp.), and a 95% confidence interval was defined for all statistical tests.

Initially, an exploratory analysis was carried out on all the variables to determine their distribution, symmetry, and the presence of outliers. The Mann-Whitney test was used to assess the difference between HDL-c, LDL-c, total cholesterol, and triglyceride values in relation to smoking and non-smoking status. The chi-square test was used to assess compliance with the LDL-c target previously established in the group of smokers and non-smokers. Statistical significance was set to p < 0.05.

## Results

From an initial population of 16,939 patients, 12,076 apparently healthy patients were included, 58.7% women and 41.3% men. A total of 4863 patients (28.7%) were excluded due to the lack of information allowing the SCORE2 to be calculated. The study population was aged between 40 and 69 years, with a mean age of 53.80±8.03 years.

In order to assess the relationship between smoking and the lipid profile of the patients, an analysis was made of the various variables under study in relation to smoking/non-smoking status.

The detailed characterization of the participants, stratified by smoking/non-smoking status, is described in Table 1.

**Table 1:** Descriptive analysis of variables by smoking/non-smoking groups

Variables (mean)	Groups	
	Smokers	Non-smokers
n	1554	10,522
Age, years	52.48 (±7.63)	53.99 (±8.03)
Males, %	67.2 (n=1044)	37.5 (n=3942)
Females, %	32.8 (n=510)	62.5 (n=6580)
HDL-c, mg/dL	51.71 (±14.36)	54.17 (±13.85)
LDL-c, mg/dL	118.89 (±34.29)	114.76 (±35.15)
Total Cholesterol, mg/dL	195.15 (±37.68)	189.60 (±38.32)
Triglyceride, mg/dL	122.73 (±61.48)	103.35 (±52.19)

The chi-squared test showed a significant difference between the percentage of female and male patients in the “smoker/non-smoker” category, with 67.2% of male smokers and 37.5% of female smokers; compared to 32.8% of male patients and 62.5% of female patients in the non-smoking group (p-value <0.001).

In terms of age distribution, the group of smokers had an average age of 52.48 years (95% CI: 52.10-52.86) and the group of non-smokers had an average age of 53.99 years (95% CI: 53.84-54.15).

To assess the distribution of the mean total cholesterol, LDL-c, HDL-c, and triglyceride values for each condition (smoker/non-smoker), the Mann-Whitney test was used, as described in Table 2.

**Table 2:** Comparison of lipid profile between smoking/non-smoking groups

		Mean, mg/dL	p-value
Total Cholesterol	Smokers	195.15 (95% CI: 193.27-197.02)	<0.001
	Non-smokers	189.60 (95% CI: 188.87-190.33)	
LDL-c	Smokers	118.89 (95% CI: 117.19-120.60)	<0.001
	Non-smokers	114.76 (95% CI: 114.09-115.43)	
HDL-c	Smokers	51.71 (95% CI: 50.99-52.42)	<0.001
	Non-smokers	54.17 (95% CI: 53.91-54.44)	
Triglyceride	Smokers	122.73 (95% CI: 119.67-125.78)	<0.001
	Non-smokers	103.35 (95% CI: 102.35-104.35)	

The test shows that the difference between cholesterol values is statistically significant, with total cholesterol, LDL-c, and triglyceride values tending to be higher in the smoking group and the HDL-c value tending to be higher in the non-smoking group.

In addition to evaluating the average cholesterol values, the authors wanted to assess whether there was a significant difference in meeting the LDL-c target set for the study. The chi-square test was used for this analysis (Table 3).

**Table 3:** Comparison of LDL-c target achievement between smoking/non-smoking groups

	LDL-c target compliance		p-value
	Accomplished, %	Not accomplished, %	
Smokers	8.0 (n=124)	92.0 (n=1430)	<0.001
Non-smokers	16.7 (n=1761)	83.3 (n=8761)	

The test shows that there is a significant difference between smokers and non-smokers when it comes to meeting the LDL-c target. In this case, a smoker has a 16.7% chance of meeting the target compared to the 8% representative of the smoker sample (p-value <0.001).

## Discussion

Smoking is one of the main cardiovascular risk factors and is a public health problem due to its high prevalence. In addition to the role of dyslipidemia as a cardiovascular risk factor, several studies have associated smoking with a worsening lipid profile, although the results are not consensual. SCORE2 is an updated risk prediction model that estimates the risk of fatal and non-fatal CVD over a 10-year period for individuals in Europe by gender, age, systolic blood pressure, total cholesterol, and smoking status. According to the result, target LDL-c values are established, which were considered in this study.

From the results obtained, there was a statistically significant difference in all cholesterol values between smokers and non-smokers, with higher total cholesterol, LDL-c, and triglyceride values in the smoking group, compared to higher HDL-c values in the non-smoking group (p-value <0.001).

These results are in line with those obtained in previous studies with other populations. In the study by Mouhamed et al. [9], conducted in a Tunisian population, lower HDL-c values and higher total cholesterol, LDL-c, and triglycerides were obtained in the group of smokers. This similarity with our study may be associated with the influence of the Mediterranean diet in both countries, which has previously been associated with an improved lipid profile [10]. A study by Nakamura et al. [11] in a Japanese population also found similar results, with higher LDL-c, total cholesterol, and triglyceride levels and lower HDL-c levels in the smoking group, which may reveal a more significant role for smoking in the lipid profile.

Not all studies have shown similar results. In the study by Moradinazar et al. [12], developed in an Iranian population, lower HDL-c and higher triglyceride values were obtained in the smoking group. However, there were no significant differences in LDL-c and total cholesterol. In the study by Momayyezi et al. [13], conducted in a Persian population, there was a higher prevalence of dyslipidemia in the smoking group, with higher triglyceride values. In contrast, the same study showed higher HDL-c values in the smoking group, as well as non-significant differences in LDL-c and total cholesterol between the groups.

When interpreting these results, we should note that different target values for total cholesterol, triglycerides, HDL-c, and LDL-c were used in the studies, with considerably higher values being used for LDL-c and triglycerides. No previous studies were found in the Portuguese population to compare lipid profile values or to assess compliance with the recommended LDL-c targets.

Regarding compliance with the LDL-c targets established for this study, according to SCORE2, there was a significant difference between the groups, with a lower percentage of compliance in the smoking group. However, we should note that, according to SCORE2, smoking status is in itself a cardiovascular risk factor, contributing to higher risk values and, consequently, lower LDL-c targets.

Despite these results, we have to consider some biases present in this study that may have influenced the results, namely the significant difference in gender distribution in both groups (with a predominance of males in the smoking group and females in the non-smoking group). It would also be important to assess the body mass index of the users under study, namely its distribution in both groups, given that overweight and obesity are a highly prevalent cardiovascular risk factor with a negative impact on the lipid profile.

The fact that the study was carried out in four different health units, which allowed for a larger sample and a population with different characteristics, is also worth highlighting. The use of a randomized sample made it possible to reduce selection bias.

## Conclusions

The researchers concluded that smoking status seems to influence the lipid profile, with higher total cholesterol, LDL-c, and triglyceride values and lower HDL-c values in the smoking group. It should be added that, in addition to higher values, smokers less often meet the LDL-c target set by the ESC/EAS 2019 guidelines. However, it would be beneficial to carry out more wide-ranging studies, particularly assessing body mass index.
